# Functional Outcome of Proximal Humerus Internal Locking System (PHILOS) Plating in Proximal Humerus Fractures

**DOI:** 10.7759/cureus.84987

**Published:** 2025-05-28

**Authors:** Abhishek Jain, Nirmesh Bhalla, Dinesh Gangwar

**Affiliations:** 1 Sports Medicine, King George Medical University (KGMU), Lucknow, IND; 2 Orthopedics and Trauma, Dr. Shyama Prasad Mukherjee (SPM) (Civil) Hospital, Lucknow, IND

**Keywords:** constant murley score, functional outcome, neer classification, philos, proximal humerus fracture

## Abstract

Background: Prognosis of proximal humerus fractures depends on the number of fracture fragments; a four-part fracture has a poorer prognosis than two or three-part fractures. The proximal humerus internal locking system (PHILOS) (DePuy Synthes, Johnson & Johnson, MA, USA) plate was designed to provide angular stability, especially in osteoporotic bones, and to decrease the high complication rates associated with these fractures. The present study is an attempt to further establish the findings and outcome of PHILOS plating in proximal humerus fractures.

Materials and methods: Around 59 patients with proximal humerus fractures were enrolled in our study, and PHILOS plating was done. Demographic details of the patient, mode of injury, type of fracture, associated injuries, and time since injury were recorded. Patients were followed at an interval of two weeks, six weeks, three months, and six months. Radiographic evaluation was done at each follow-up to assess fracture union and any complications. Functional outcome was assessed using the 100-point Constant-Murley scoring system.

Observation and results: According to Neer’s classification, there were 22 (37.3%) patients each classified as type 2 and 3, while the remaining 15 (25.4%) were classified as type 4. The mean Constant-Murley score was 53.88±12.31 at first follow-up as compared to 71.25±14.69 at final follow-up, thereby showing an increase of 17.37±4.62. At final follow-up, functional outcome was rated as very good, good, fair, and poor in 12 (20.3%), 22 (37.3%), 16 (27.1%), and 9 (15.3%) cases, respectively.

Conclusion: The present study showed low post-operative complication rates and sustainable improvement in the functional status of patients in subsequent follow-ups after PHILOS plating.

## Introduction

Proximal humerus fractures occur around the surgical neck of the humerus. It represents 5-6% of all fractures and is the seventh most frequent fracture among adults [1]. Females are more prone to proximal humeral fractures than males [2]. Higher incidence of proximal humeral fractures among older populations and females is associated with compromised bone density. Most proximal humerus fractures are minimally displaced, low-energy osteoporotic fractures and are effectively treated with conservative treatment [3]. A bimodal distribution pattern of proximal humerus fractures has been reported, high-energy injuries in younger patients (motor vehicle accident) and low-energy injuries in older patients who are possibly at risk of osteoporosis [4]. Proximal humerus fractures in older patients are associated with aging musculature, rotator cuff tendinopathy, shoulder arthropathy, and various comorbid conditions.

About 80% of proximal humeral fractures are undisplaced or minimally displaced fractures that can be effectively treated with conservative treatment [3]. Options of closed reduction with K-wire fixation, open reduction with plating, and newer intramedullary devices addressing periarticular fractures are also available. Non-operative treatment and fixation using K-wires lead to stiffness and decreased range of motion. According to Neer’s classification, a four-part fracture has a poorer prognosis than two- or three-part fractures. The proximal humerus internal locking system (PHILOS) plate was designed to provide angular stability, especially in osteoporotic bones, and to decrease the high complication rates associated with these fractures [5].

These plates have a low profile and are biomechanically better suited for the fixation of proximal humerus fractures. They provide angular stability and locking screw anchorage in weak osteoporotic bones. This plate also provides multiple locking screw options, which can be inserted in a convergent/divergent fashion in the humeral head for better pull-out strength. These implants can withstand the physiological loads (muscular force) in osteoporotic bone. Highly complex three- and four-part fractures can be reconstructed with rotator cuff sutural ties through the holes in the plate, thereby enhancing the functional outcome. The present study is an attempt to further establish the findings and outcome of PHILOS plating in proximal humerus fractures.

## Materials and methods

The study design was prospective-observational and was conducted in the Department of Orthopedic Surgery, Dr. Shyama Prasad Mukherjee (Civil) Hospital, Hazratganj, Lucknow. Patients presenting with proximal humerus fractures to the Outpatient Department of Orthopedics and Emergency Department, fulfilling inclusion criteria, were enrolled in the study from July 2023 to June 2024. The study was approved by the Institutional Ethics Committee.

All the patients above 18 years of age with closed proximal humerus fractures and injury within six weeks were included, except those with pathological fractures other than osteoporosis, patients not giving consent, or not fit for surgery.

The sample size was calculated using the formula: $n = \frac{Z^2 \cdot p \cdot (1 - p)}{d^2}$, where n=sample size; z=statistic for a level of confidence (for the level of confidence of 95%, which is conventional, the z value is 1.96); p=expected prevalence of proportion (in proportion of one, if 4%, p=0.04) (Kishore and Tonape, 2020) [6]; d=precision (in proportion of one; if 5%, d=0.05 n=1.96*1.96*0.04*0.96/(0.05)2 (n=59))

**Figure 1:**
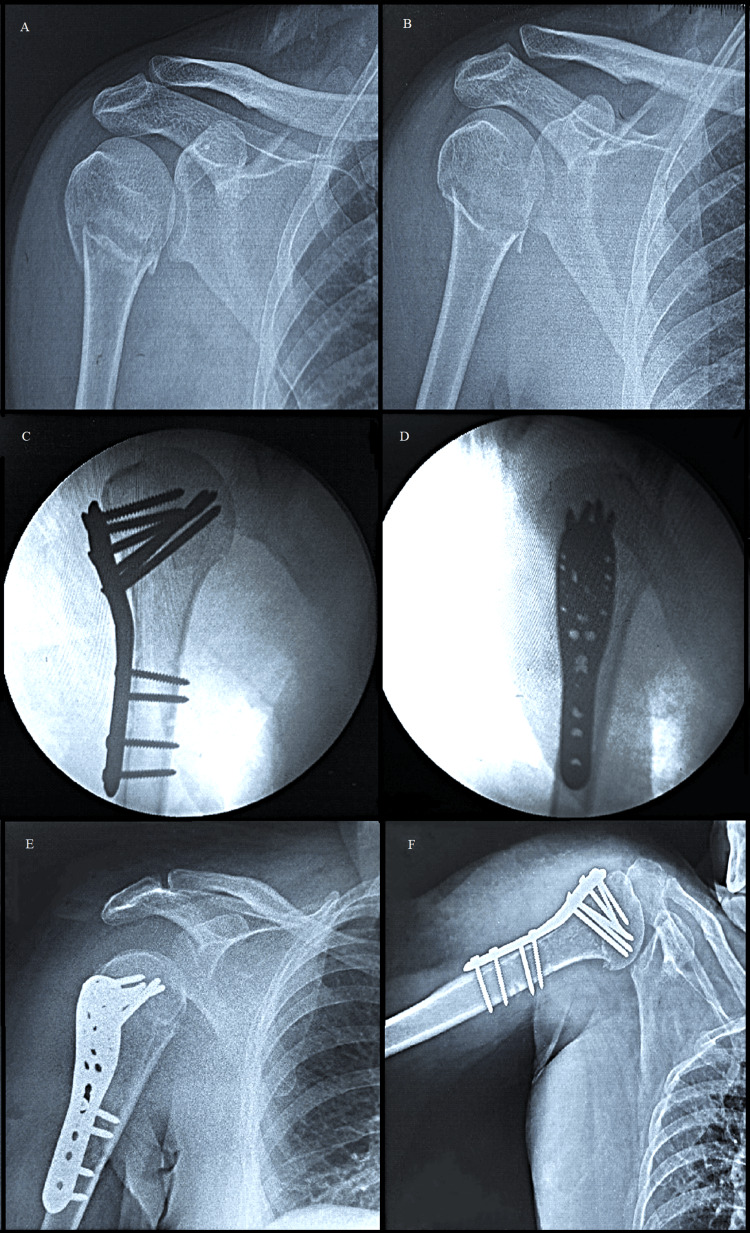
Open reduction and internal fixation of proximal humerus fracture (Neer type 2) with PHILOS plate in a 63-year-old female A: preoperative radiograph of the shoulder in AP view; B: preoperative radiograph of shoulder in the lateral view; C: postoperative C-arm image of shoulder in AP view; D: postoperative C-arm image of shoulder in the lateral view; E: postoperative radiograph of shoulder in the lateral view at the second follow-up; F: postoperative radiograph of shoulder in the axillary view at the second follow-up PHILOS: proximal humerus internal locking system; AP: anteroposterior

A total of 59 patients were enrolled in the study after giving written informed consent. The data were collected using a semi-structured questionnaire (Appendix 1). Fracture type was classified using Neer’s classification. Patients were treated by open reduction and internal fixation with a PHILOS plate (Figure 1).

Surgery was performed using the deltopectoral/deltoid splitting approach in a supine position. Pre- and post-operative radiographic evaluation was done. Functional outcome was assessed using the 100-point Constant-Murley scoring system. Patients were followed at an interval of two weeks, six weeks, three months, and six months. Radiographic evaluation was done at each follow-up to assess fracture union and any complications.

Statistical analysis

Data was analyzed using the IBM SPSS Statistics for Windows, Version 21 (Released 2012; IBM Corp., Armonk, New York, United States). Continuous data were presented as mean ± standard deviation (SD) and assessed by paired t-test, Wilcoxon signed rank test, chi-square test, and ANOVA test, and the Tukey-HSD test was used for comparison between the means of parameters. A p-value less than 0.05 is considered statistically significant.

## Results

The age of patients ranged from 24 to 71 years; the mean age of patients was 47.29±13.62 years. Male dominance was observed in the study population (67.8%); approximately one-third (32.2%) of the patients were female. The mode of injury in the majority of the patients was a road traffic accident (59.3%); in the rest of the patients, the cause of injury was a fall against the ground (23.7%) and a fall from height (16.9%). Shoulder dislocation was found to be associated with proximal humerus fracture in only six (10.2%) patients. On Neer’s classification, types 2 and 3 (37.3% each) were more common; the remaining 25.4% of fractures were classified as type 4. The time lag between injury and surgery ranged from four to 26 days; the mean duration was 11.95±5.35 days. Surgery for maximum patients was done within 15-21 days of injury (37.3%), followed by eight to 14 days (27.1%). Only 15.3% of patients underwent surgery in ≤7 days, and 20.3% underwent surgery after 21 days of injury.

The deltoid split (DS) approach for treatment was adopted for 30 (50.8%) patients; in the rest, 49.2% of patients adopted the delto-pectoral (DP) approach. The duration of surgery ranged between 70 and 150 minutes; the mean duration of surgery was 108.31±19.58 minutes.

**Figure 2:**
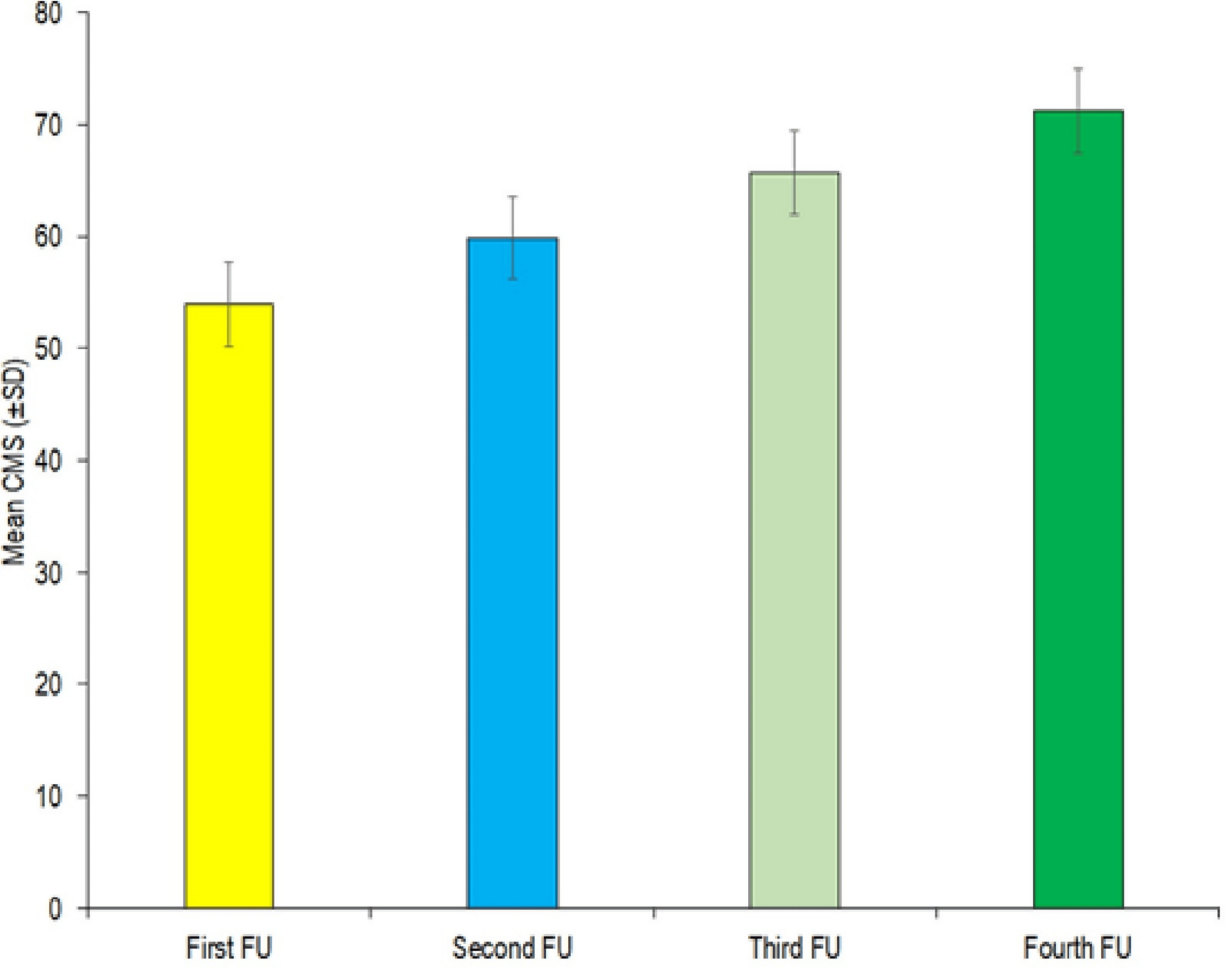
Constant-Murley score of the study population at different follow-up First follow-up done at two weeks; second follow-up done at six weeks; third follow-up done at three months; fourth follow-up done at six months

On the first follow-up, the Constant-Murley score (CMS) ranged from 23 to 72; the mean CMS was 53.88±12.31. On the second follow-up, CMS ranged from 27 to 78; the mean CMS was 59.88±13.20. An increment of 6.00±2.55 in the first follow-up CMS was observed; the change was 11.14% and was statistically significant (Table 1). On the third follow-up, CMS ranged from 33 to 85, and the mean CMS increased further to 65.71±13.75. A change of 11.83±3.41 in the first follow-up CMS was observed; the change was quantified as 21.96% of the first follow-up. This change was found to be statistically significant. On the fourth follow-up, CMS ranged from 35 to 92; the mean CMS was 71.25±14.69. A change of 17.37±4.62 in the first follow-up CMS was observed; this change was 32.24% of the first follow-up CMS; this change was also statistically significant. Figure 2 shows CMS at each follow-up.

**Table 1:** Change from the first follow-up Constant-Murley score to subsequent follow-ups (paired ‘t’ test)

Details	Mean change	SD	% Change	‘t’	‘p’
Second follow-up	6.00	2.55	11.14	18.05	<0.001
Third follow-up	11.83	3.41	21.96	26.65	<0.001
Fourth follow-up	17.37	4.62	32.24	28.89	<0.001

CMS <56 was considered a poor outcome, 56-70 as a fair outcome, 71-85 as a good outcome, and 86-100 as a very good outcome. At the first follow-up, 23 (39.0%) patients had poor outcomes, 33.9% had fair outcomes, 27.1% had good outcomes, and none had very good outcomes. At the second follow-up, 22 (37.3%) had poor outcomes, 20 (33.9%) had fair outcomes, 17 (28.8%) had good outcomes, and none had very good outcomes. Change in outcome from that at first follow-up was not found to be statistically significant. At the third follow-up, 12 (20.3%) patients had poor outcomes. Fair, good, and very good outcomes were observed in 35.6%, 42.4%, and 1.7%. A statistically significant change in the outcome of the first follow-up was observed at the third follow-up. At the fourth follow-up, only nine (15.3%) had poor outcomes. Fair, good, and very good outcomes were observed in 27.1%, 37.3%, and 20.3%. A statistically significant change in the outcome of the first follow-up was observed at the third follow-up (Table 2). The outcome at the fourth follow-up was considered final.

**Table 2:** Assessment of outcome at different follow-ups (based on Constant-Murley score (CMS)) Wilcoxon signed rank test (comparison of first follow-up (FU) with other follow-ups)

SN	Outcome (CMS)	1st FU		2nd FU				3rd FU				4th FU			
		No.	%	No.	%	z	p	No.	%	z	p	No.	%	z	p
1	Poor (<56)	23	39.0	22	37.3	1.414	p=0.157	12	20.3	4.690	p<0.001	9	15.3	6.206	p<0.001
2	Fair (56-70)	20	33.9	20	33.9			21	35.6			16	27.1		
3	Good (71-85)	16	27.1	17	28.8			25	42.4			22	37.3		
4	V. good (86-100)	0	0.0	0	0.0			1	1.7			12	20.3		

**Figure 3:**
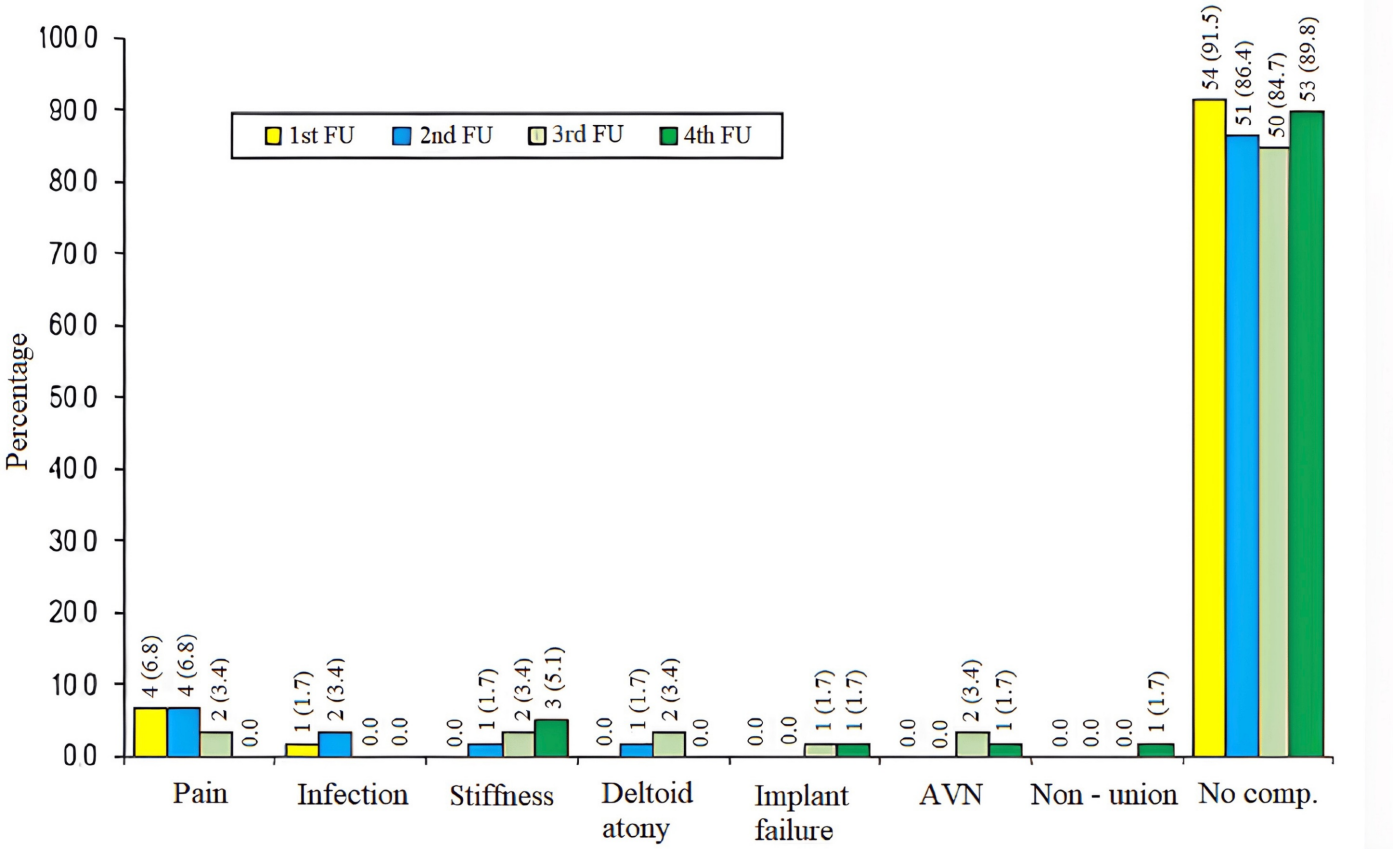
Post-operative complaints/complications at different follow-ups (total patients=59) First follow-up is done at two weeks; second follow-up is done at six weeks; third follow-up is done at three months; fourth follow-up is done at six months. AVN: avascular necrosis

Post-operative complaints/complications at different follow-ups are shown in Figure 3. On the first follow-up (at two weeks), infection was reported in one (1.7%) patient, and four (6.8%) patients had complaints of pain; the rest (91.5%) of the patients did not report any complaints or complications at the first follow-up. On the second follow-up (at six weeks), the majority of the patients (86.4%) did not report any complaint or complication. Pain, infection, stiffness, and deltoid artery damage were reported in 6.8%, 3.4%, 1.7%, and 1.7%, respectively. On the third follow-up (at three months), the majority of patients (84.7%) did not report any complaint or complication. Pain, stiffness, deltoid atony, implant failure, and avascular necrosis were reported in 3.4%, 3.4%, 4%, 1.7%, and 3.4% of patients, respectively. On the fourth follow-up (at six months), the majority of patients (89.8%) did not report any complaint or complication. Stiffness was the most common complication (5.1%); each of the following complications (implant failure, avascular necrosis, and non-union) was reported by one (1.7%) patient.

The majority of patients aged ≤45 years had good to very good outcomes (75%), while the majority of patients aged 46-60 years and >60 years had poor to fair outcomes (61.1% & 53.9%). This difference was found to be statistically significant (c²=13.849 (df=6); p=0.031).

A good to very good outcome was observed in a higher proportion of patients with type 2 and type 3 fractures as compared to type 4 fractures (54.5% and 72.8% vs. 40.0%), but this difference was not found to be statistically significant (c²=6.841 (df=6); p=0.336).

The difference in the outcome of patients managed with different treatment approaches was not found to be statistically significant (c²=7.680 (df=3); p=0.053).

The proportion of patients with a good to very good outcome was higher for those whose duration of surgery was up to 90 min (66.7%) and 91-120 min (57.1%) as compared to those with >120 min (40.0%); yet this difference was not found to be statistically significant (Table 3) (c²=7.258 (df=6); p=0.298).

**Table 3:** Association of the duration of surgery (in minutes) with different outcomes F=2.728 (ANOVA); p=0.053

Final outcome	N	Min.	Max	Mean	SD
Poor	9	90.0	150.0	123.33	15.81
Fair	16	70.0	150.0	105.00	20.66
Good	22	90.0	150.0	108.64	17.81
V. good	12	80.0	150.0	100.83	19.75
Total	59	70.0	150.0	108.31	19.58

The mean duration of surgery of patients with poor outcomes was maximum (123.33±15.81 min), and that of very good outcomes was minimum (100.83±19.75 min). The duration of surgery of patients with fair and good outcomes was 105.00±20.66 min and 108.64±17.81 min, respectively. A difference in the duration of surgery of poor-outcome and very-good-outcome patients was found to be statistically significant (Table 4).

**Table 4:** Comparing mean values of duration of surgery (in minutes) among different outcomes (Tukey HSD test)

Outcome	Mean diff.	SE	‘p’
Poor Vs. Fair	18.33	7.82	0.100
Poor Vs. Good	14.70	7.42	0.208
Poor Vs. V. good	22.50	8.27	0.042
Fair Vs. Good	-3.64	6.16	0.935
Fair Vs. V. good	4.17	7.16	0.937
Good Vs. V. good	7.80	6.73	0.655

## Discussion

Various surgical techniques are present to manage the proximal humerus fracture, which include closed reduction and percutaneous pinning, open reduction, nailing, hemiarthroplasty, conventional plate, and advanced locking plate, but none of the above surgical techniques has proven to be ideal. Therefore, the present study was planned as an observational study to analyze the functional outcome of the PHILOS plating in proximal humerus fractures. Observational design has been adopted by most of the researchers who dealt with proximal humerus fractures using PHILOS plating; a small number of patients had been included by Rose et al. (n=16) [7], She et al. (n=19) [8], and Charalambous et al. (2007) [9] (n=23), while a larger number of patients had been included by Sahu et al. (n=108) [10] and Xiong et al. (n=112) [11]. In the present study, 59 patients have been included; an almost similar number of patients had been included by Kumar et al. (n=51) [12], Jacob and Nayak (n=42) [13], and Kishore and Tonape (n=53) [6]. Studies with larger sample sizes are considered to be more reliable, but the duration of the study period, density of the population, hospital set-up, hospital attendance, and various other factors are barriers to larger sample sizes.

In the present study, all the patients were clinically examined, and the type of fracture was classified using Neer’s classification. Though Codman Classification [14] and the AO/ASIF system [15] are also used to describe the type of proximal humerus fractures, Neer’s classification is the most popular. In the present study, patients were followed at two weeks, six weeks, three months, and six months, and the outcome of PHILOS plating was assessed using CMS. These scores are based on the level of pain, activities of daily life, range of motion, and power and are capable of assessing the functional status of the patient. Apart from it, the Disability of Arm, Shoulder and Hand (DASH) score [16,17] and the Paavolainen method [17] have also been used for the assessment of post-operative outcomes.

The age of patients included in the present study ranged from 24 to 71 years (mean 47.29±13.62 yrs), with the maximum patients in the younger age group ≤45 years (47.5%), and only 22% were >60 years. On comparing the average age of patients enrolled in the contemporary studies, older patients (average age ≥60 yrs) had been part of the research studies by Koukakis et al. [18], Charalambous et al. [9], Shahid et al. [19], Xiong et al. [11], and Zhao et al. [20]. The maximum average age of patients was observed for the participants of the study by She et al. (mean 82.52; range 75-87 years). [8]. Younger patients (average age ≤40 yrs), as compared to the present study, had been included by Kumar et al. [12] and Aliuddin et al. [21]. Pandya and Soni (mean 53.7 yrs) [22], Doshi et al. (54.3±5.8 yrs) [23], Narayanan and Balasubramanian (58.8 yrs) [24], Kumar et al. (mean 58 yrs) [12], Spolia et al. (mean 48.4 yrs) [25], Ethiraj et al. (mean 46.8 yrs) [26], and Jhamnani et al. (mean 54.5±64 yrs) [27]. Clark et al. (2017) [28] had supported that biological processes involved in fracture healing are affected by age.

In the present study, only 25.4% of patients were type 4 on Neer’s classification; 37.3% of patients each were of type 2 and type 3. In most of the contemporaries, the proportion of type-4 fractures was close to the present study. However, on comparing with the present study, a higher proportion of type 4 fractures was reported by Abdelrahman et al. (55%) [29] and Kumar et al. (45.1%) [12]. A very low proportion of type 4 fractures had been reported by Rose et al. (12.5%) [7], Nourozi et al. (10.8%) [30], Sahu et al. (0.0%) [10], and Ethiraj et al. (10.0%) [26].

At the initial follow-up, pain and infection were reported in 6.8% and 1.7% of patients; at the second follow-up, pain, infection, stiffness, and deltoid atony were reported by 6.8%, 3.4%, 1.7%, and 1.7% of patients. At the final follow-up, only six (10.2%) patients had complications of stiffness (5.1%), implant failure (1.7%), avascular necrosis (1.7%), and non-union; the rest of the 89.8% of patients were treated without any complications. On comparing the rate of complications in the contemporary studies with the present study, the complication rate was very close in studies by Kumar et al. (85.8%) [12], Jacob and Nayak (81.0%) [13], and Spolia et al. (83.3%) [25]. The complication rate was found to be higher than in the present study in studies by Charalambous et al. (36.0%) [9], Rose et al. (25.0%) [7], Norouzi et al. (35.1%) [30], Pandya and Soni (39.0%) [22], Ethiraj et al. (25.0%) [26], and Jhamnani et al. (34.4%) [27]. On the other hand, no postoperative complication was observed by Abdelrahman et al. [29] and by Narayanan and Balasubramanian [24], and a very low complication rate was observed by Shahid et al. (4.2%) [19], Sahu et al. (5.6%) [10], and Xiong et al. (8.9%) [11].

In the present study, the outcome of patients was assessed using CMS at all the follow-ups. At the first follow-up, CMS was 53.88±12.31, which sequentially increased to 71.25±14.69 at the fourth follow-up. Average CMS scores of 77 had been reported by Abdelrahman et al. [29] and 79.0 by Kumar et al. [12], which were close to that observed in the present study. A significant change in the first follow-up CMS score was observed at the second, third, and fourth follow-ups, which indicates sequential functional improvement. Categorically, the majority of patients at the first three follow-ups had poor to fair improvement in functional outcome (72.9%, 71.2%, and 55.9%), while at the fourth (final) follow-up, the majority of patients had good to very good functional outcomes (57.6%: 37.3% good and 20.3% very good), and only 15.3% of patients had poor outcomes.

Better results as compared to the present study have been reported by Sahu et al. [10], Kumar et al. [12], Narayanan and Balasubramanian [24], Pandya and Soni [22], Spolia et al. [25], Jhamnani et al. [27], and She et al. [8], while the results of Ethiraj et al. [26] were close to our study. Only Jacob and Nayak [13] showed poorer results as compared to the present study. Differences in functional outcome can be attributed to differences in assessment time, proportional differences in mode of injury, older aged patients, gender, comorbid conditions, and infrastructural facilities available at the institutions.

In this study, an attempt has been made to find out the association of functional outcome with demographic and clinical factors, and a significant association of functional outcome with younger age only. Other factors such as mode of injury, incidence of shoulder dislocation, type of fracture (Neer’s classification), surgical approach, time lag in injury to surgery, and duration of surgery did not show any significant association. Only a few studies have commented on this. Aliuddin et al. [21] had found significantly lower DASH scores of younger patients, while Koukakis et al. [18] did not find any significant difference in the functional outcome of younger and older patients.

Limitations of the study

The effectiveness of PHILOS on other management techniques could not be commented on, as we included proximal humerus fractures managed by the PHILOS technique only. Studies with larger sample sizes are considered to be more reliable, but the duration of the study period, the density of population, hospital set-up, hospital attendance, and various other factors are barriers to larger sample sizes. Hence, there is a need for multicentric and meta-analyses for the generalized data.

## Conclusions

The present study showed that improvement in outcome at first follow-up (two weeks) was observed on subsequent follow-ups at six weeks, three months, and six months. Its effectiveness is not compromised due to other demographic and clinical factors. There is a significant association of younger age with a good-very good outcome; the role of other factors in the outcome could not be established. Low post-operative complication rates and sustainable improvement in the functional status of patients were observed. The effectiveness of PHILOS on other management techniques could not be commented on, as we included proximal humerus fractures managed by the PHILOS technique only. Further studies on a larger sample size should be carried out to evaluate the role of other clinicodemographic factors on the functional outcome.

## Figures and Tables

**Figure 4:**
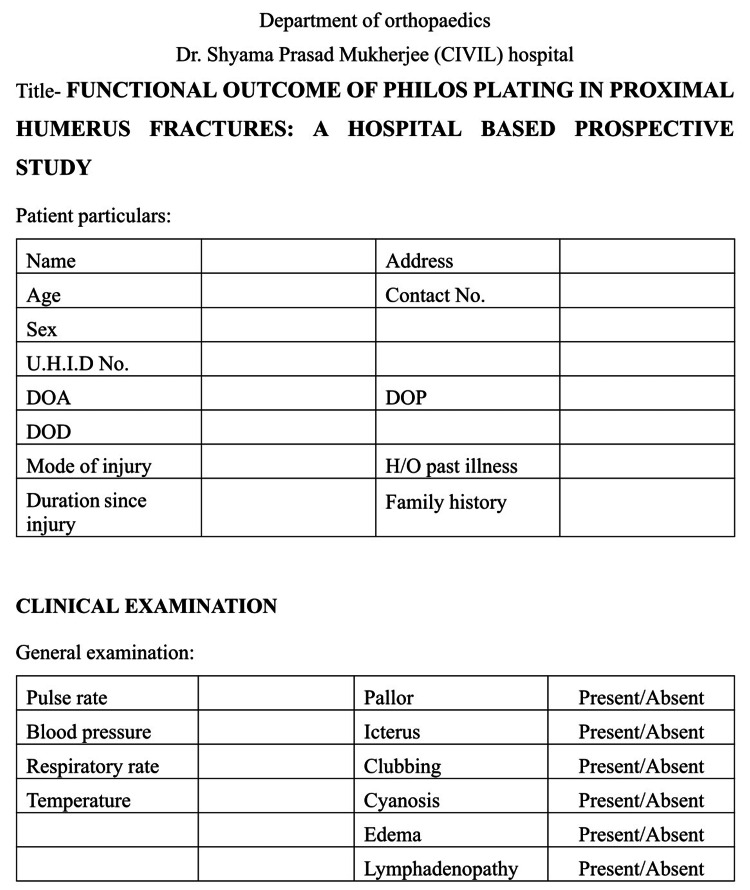
Questionnaire (page 1)

**Figure 5:**
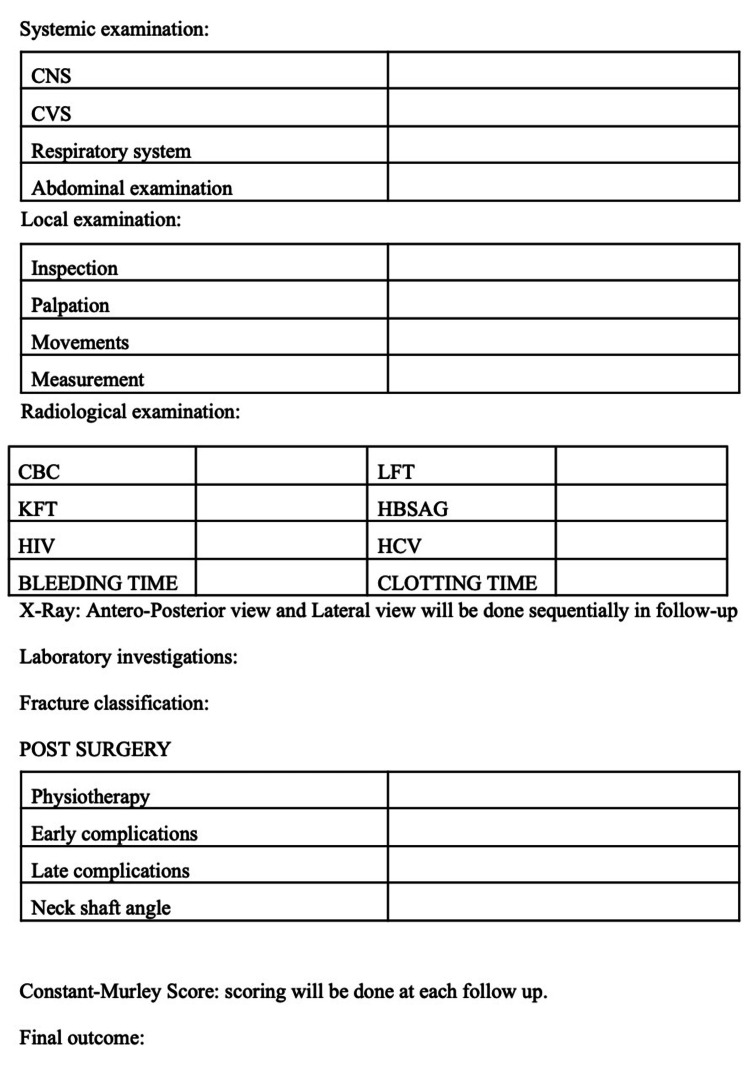
Questionnaire (page 2)

**Figure 6:**
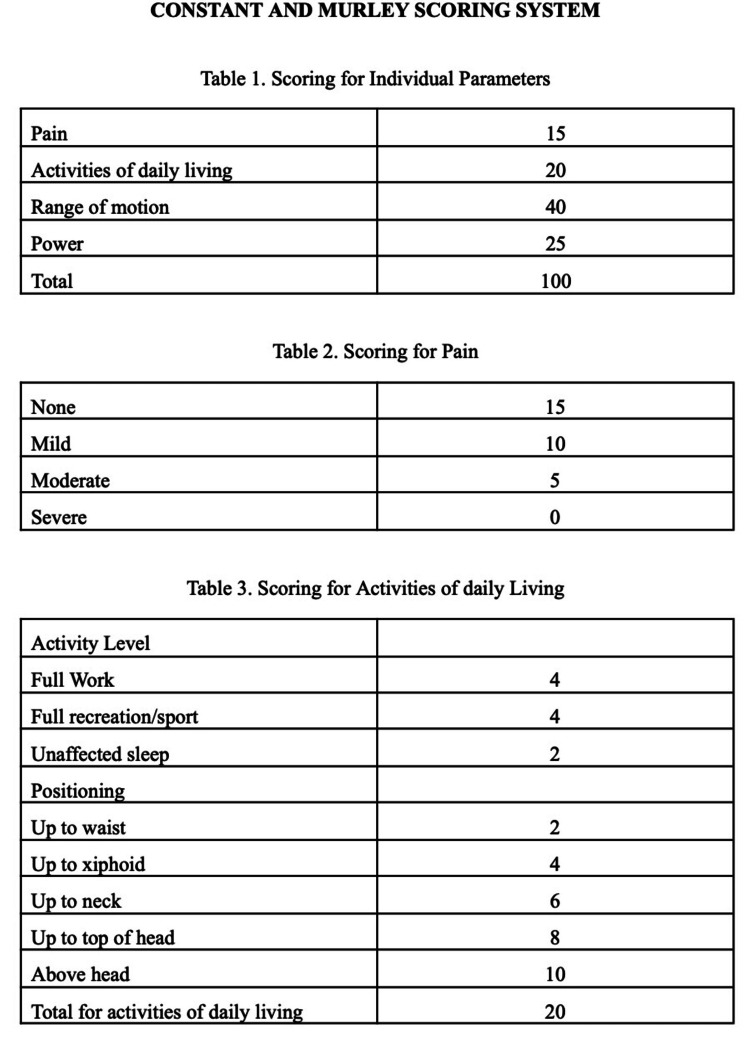
Questionnaire (page 3)

**Figure 7:**
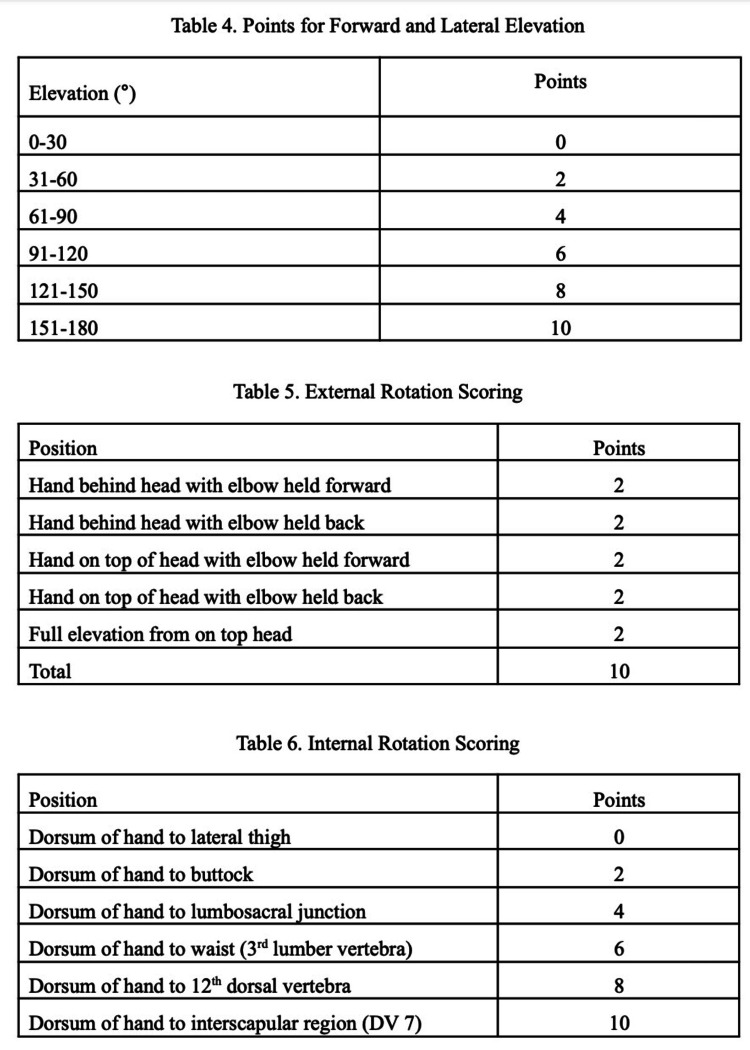
Questionnaire (page 4)

**Figure 8:**
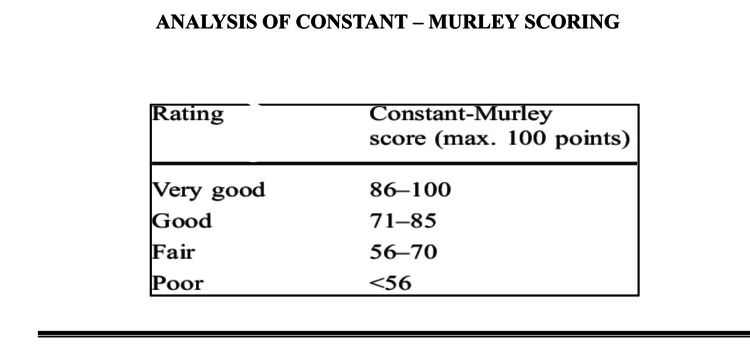
Questionnaire (page 5)

## Appendices

Appendix 1
